# Navigating the Nexus of Bioethics and Geopolitics: Implications for Global Health Security and Scientific Collaboration

**DOI:** 10.1007/s11673-024-10379-3

**Published:** 2024-08-20

**Authors:** Alexandra Klimovich-Mickael, Mariusz Sacharczuk, Michel Edwar Mickael

**Affiliations:** 1 PM Research Center, Väpnaregatan 22, 58649 Linköping, Sweden; 2 Institute of Genetics and Animal Biotechnology of the Polish Academy of Sciences, Ul. Postepu 36A, 05-552 Jastrzębiec, Magdalenka Poland

**Keywords:** Bioethics, Viruses, Geopolitics, Public health

## Abstract

Bioethics plays a pivotal role in guiding ethical decision-making within the realm of medical research and healthcare. However, the influence of geopolitics on bioethical considerations, particularly regarding bioweapons research, remains an underexplored area. This study delves into the uncharted territory of how international political interests can intersect with bioethical principles, potentially shaping collaborative efforts and global health policies related to bioweapons research. Through a hypothetical scenario involving a hypothetical pathogen, a collaborative effort between unspecified countries, we examine the implications of such cooperation on global health governance, with a specific focus on bioweapons research. Ethical dilemmas surrounding responsible research, potential risks and benefits, equitable distribution of findings, and biosafety measures are explored. This analysis underscores the importance of transparent and responsible practices in bioweapons research amidst geopolitical tensions. By striking a balance between national interests and international solidarity, we advocate for robust bioethical frameworks to navigate such collaborations for the collective well-being of humanity and to mitigate potential risks associated with bioweapons research.

## Introduction

The potential consequences of usages of biological weapons are severe, and historical examples highlight the threats involved. For instance, during World War II, Unit 731 of the Imperial Japanese Army conducted extensive experiments with biological agents on human subjects, leading to the development and the release of plague-infected fleas over Chinese cities (Li 2017; Johnson 2022; Roberts 2012). Similarly, the Soviet Union’s secret biological weapons programme, known as “Biopreparat,” involved the production and stockpiling of large quantities of anthrax and smallpox, raising concerns about the potential for unintended release or misuse (Roffey 2015). In more recent times, the Amerithrax attacks in 2001 demonstrated the impact of bioterrorism. Letters containing anthrax spores were sent to various locations in the United States, causing several deaths and widespread panic (Anderson and Bokor 2012). These events highlighted the challenge imposed by the development of biological weapons and the risks associated with it (Nelson et al. 2019).

To address these issues, the Biological Weapons Convention (BWC) treaty was ratified in 1972 (Huigang et al. 2022). This agreement explicitly prohibits the development, production, stockpiling, acquisition, or retention of biological and toxin weapons (Kadlec et al. 1997). This treaty, which came into force in 1975, marked the first international agreement to ban an entire category of weapons (Sims 2007; Meng 2017). Its purpose is to prevent the use of biological agents for hostile purposes, thereby promoting global security and reducing the risk of bioterrorism and biological warfare (Wissinger 2015). The BWC has been ratified by over 180 states, reflecting a broad consensus on the dangers posed by biological weapons (Johnson 2024). The treaty defines biological weapons as those that use biological agents, such as bacteria, viruses, fungi, or their toxins, with the intent to cause harm to humans, animals, or plants. By banning these weapons, the BWC seeks to promote the peaceful use of biological research and technology while discouraging their use in warfare or terrorism.

However, current regulatory frameworks for bioweapons research, like the Biological Weapons Convention (BWC), do not explicitly address the influence of geopolitics on the development and oversight of biological weapons. Geopolitical factors, including international relations, strategic alliances, and diplomatic tensions, can significantly impact transparent data sharing, information dissemination, and global collaboration, thereby necessitating their consideration in the ethical evaluation of bioweapons research. By incorporating geopolitics into these regulatory frameworks, we can promote a more comprehensive approach, encouraging responsible research practices, transparent communication, and cross-border cooperation to better manage the risks associated with bioweapons development.

In this research article, we explore a hypothetical scenario where two powerful and technologically advanced countries, locked in a covert power struggle, collaborate on the knowledge and techniques for developing bioweapons involving engineered pathogens. We examine the ethical implications of this collaboration, focusing on how geopolitical factors and international tensions can impact the responsible conduct of such research. By analysing the potential risks and benefits, the implementation of biosafety and biosecurity measures, and the transparency of communication between these countries, we uncover the intricate dynamics of international scientific cooperation in the context of bioweapons. Through this analysis, we aim to emphasize the significance of integrating geopolitics with bioethics in overseeing bioweapons research. The study underlines the importance of establishing robust regulatory frameworks that address the unique challenges posed by bioweapons, ensuring that international collaboration promotes the responsible use of scientific knowledge for the collective security of humanity. By addressing these issues, we aim to foster a more secure global environment where scientific advancements in the biological domain are directed toward peaceful and ethical purposes.

## Methods and Hypothesis

This study aims to investigate the hypothetical motivations and potential reasons why two powerful countries might engage in knowledge transfer related to bioweapons development. We assume that Country A, with a larger GDP and greater financial resources for defence and military research, may have strategic interests in collaborating with Country B, known for its rapid economic growth, large population, and advanced biotechnology capabilities. To explore these hypothetical motivations, this study uses speculative scenarios and fictional narratives to provide insights into the potential drivers behind knowledge transfer in bioweapons research between powerful countries. The analysis will focus on the broader geopolitical context and the potential consequences of such collaborations on global security and ethical considerations. It will emphasize the importance of responsible research practices and the role of international scientific cooperation in managing risks related to biological weapons, with a particular focus on the prevention of bioterrorism and other threats to global stability.

## Results

### Country A Motivations

**Fig. 1:**
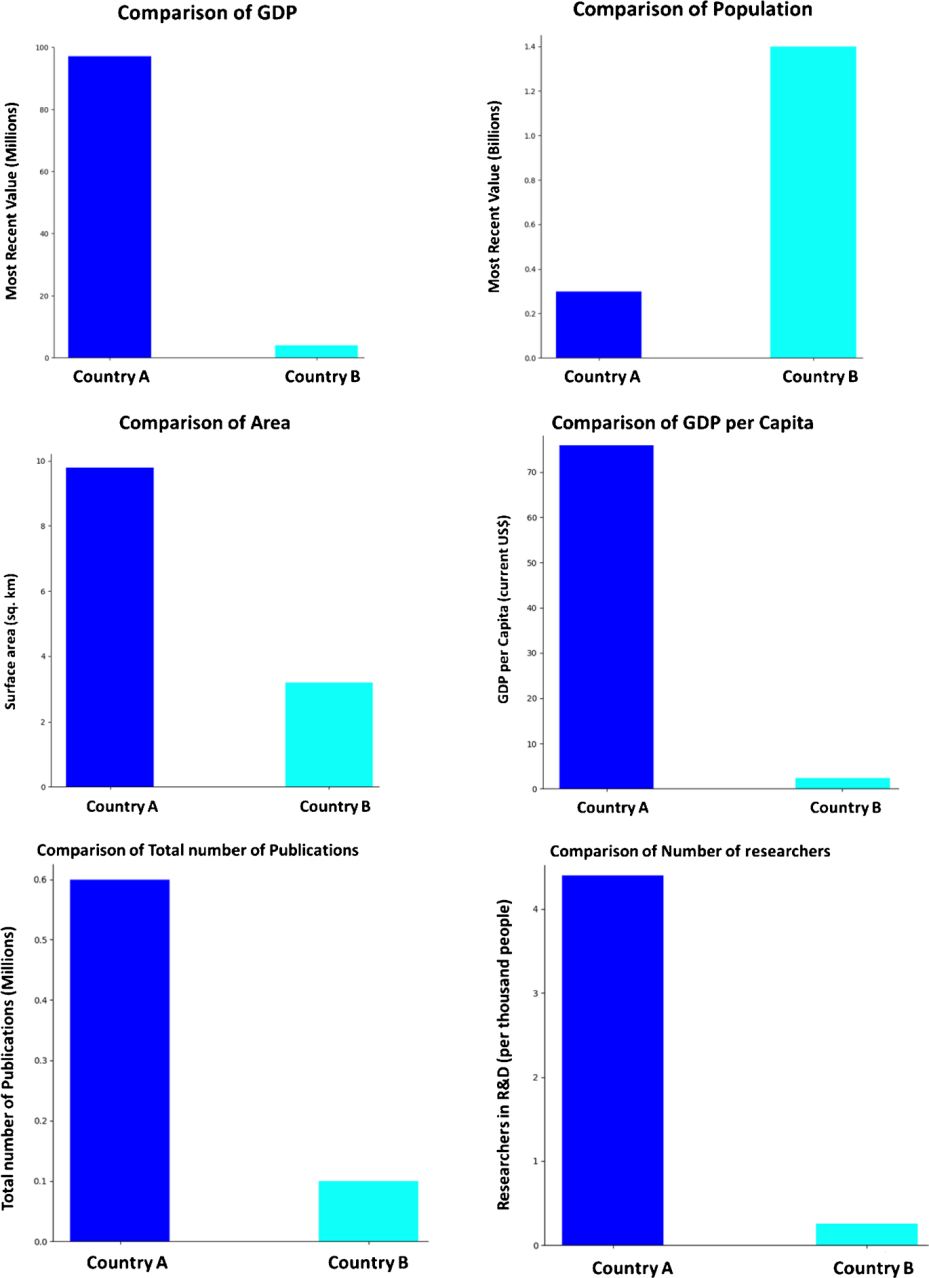
Comparison between the two hypothetical countries

Within this fictional context, we explore the reasons behind Country A’s decision to fund Country B’s research and development of bioweapons. Given that these countries possess distinct socio-economic strengths and are engaged in a covert power struggle, we aim to illuminate the possible motivations and underlying factors driving this knowledge transfer (Fig. 1). By presenting various hypothetical points, we delve into the geopolitical, strategic, and ethical considerations that could shape this collaboration on bioweapons development. Through this analysis, we aim to provide insights into the complexities of international scientific cooperation in the realm of bioweapons, while maintaining a critical perspective on the ethical ramifications and implications for global security.

In order to gain insight into real-world bioethical conflicts due to geopolitics, we made a comparison of the research abilities of America (Country A) and India (Country B).

In this speculative scenario, Country A might opt to fund Country B’s research and development of bioweapons to create a relationship of dependency. By providing financial resources and expertise, Country A can foster a sense of obligation and reliance in Country B, allowing Country A to exert significant influence over Country B’s decisions and strategic direction, according to the Power and Dependency Theory [13]. This dependency allows Country A to guide Country B’s policies, aligning them with its own geopolitical objectives. Through this dependency, Country A can manipulate the course of research, ensuring it serves its interests, whether to gain a competitive advantage or to maintain a favourable balance of power. As Country B becomes increasingly reliant on Country A’s funding and resources, Country A can dictate terms, steer policy discussions, and shape strategic partnerships to its advantage. This could extend to other areas, such as trade, diplomacy, or regional alliances, where Country A’s influence might grow as Country B’s dependency deepens. However, the power imbalance created by such dependency could also foster resentment or backlash, with Country B seeking alternative allies or secretly developing its own capabilities to regain autonomy.

By funding Country B’s bioweapons research, Country A can gather critical intelligence on bioweapon development and testing. This support gives Country A insights into how bioweapons can be used, both offensively and defensively, which is crucial for military planning and strategic decision-making. A historical precedent for this kind of intelligence-gathering is Operation Paperclip. After World War II, the United States recruited German scientists, engineers, and technicians, including those involved in Nazi Germany’s chemical and biological weapons programmes. This allowed the United States to acquire valuable expertise in advanced technologies.

Country A’s funding of Country B’s bioweapons research could reflect a strategic interest in the biotechnology and pharmaceutical sectors, with an eye toward significant economic returns. By financially supporting Country B, Country A could secure rights to new intellectual property, potentially gaining a competitive advantage in the global biotech market. This arrangement might allow Country A, through partnerships with major pharmaceutical companies, to tap into a range of new products and technologies. Big Pharma could play a central role in this dynamic, capitalizing on Country A’s access to bioweapons-related research. By collaborating with Country B, these companies might secure early access to ground-breaking innovations, which they can commercialize in various forms—such as new vaccines, drugs, or biotechnology-based treatments. This commercialization could yield substantial profits for these pharmaceutical giants while bolstering Country A’s economic interests. However, this scenario also raises concerns about equity and global health. If Country A, in collaboration with Big Pharma, monopolizes the research outcomes, it could restrict access to these technologies for countries with lower incomes (Nealey et al. 2015). The high costs of these innovations might put them out of reach for developing nations, leading to disparities in healthcare and creating a divide between wealthier and poorer countries in terms of medical technology access.

Country A’s funding of bioweapons research in Country B may signal a broader strategic alliance between the two nations, aiming to strengthen diplomatic ties and pursue shared goals. This partnership could be designed to foster cooperation on geopolitical and security issues. By investing in Country B’s research, Country A may be signalling a commitment to the alliance, demonstrating its support through financial backing and technology transfer. This strategic alliance might lead to joint research projects, coordinated security efforts, and even a broader geopolitical network that aligns with Country A’s interests. However, strategic alliances that focus on bioweapons carry significant risks. If the partnership breaks down or one party uses the research for malicious purposes, it could lead to destabilization and heightened security threats. This risk was highlighted during the turmoil following the fall of the Soviet Union, when the disintegration of the Soviet alliances resulted in the leakage of nuclear secrets and weapons, creating serious global security concerns (Akhtar 2023)[17]. Also as Country A funds and transfers knowledge to Country B, it loses control over how those bioweapons are used (Ben and Ouagrham-Gormley 2012). Country B could deploy them in ways that counter Country A’s intentions, leading to unintended consequences. Second, if other international players learn about these collaborations, they might start their own bioweapons research, potentially leading to an arms race.

The country A strategy of employing Country B as a proxy to pursue its strategic interests without direct involvement allows Country A to minimize its own direct exposure, potential international repercussions, and maintain a degree of plausible deniability (Zellman 2014). If the research were to face international condemnation or cause unforeseen consequences, Country A could distance itself from the fallout, placing the burden of responsibility on Country B. This insulation from direct involvement helps Country A avoid diplomatic crises, sanctions, or other repercussions that might result from being linked to controversial bioweapons activities. However, the lack of direct oversight and accountability in proxy arrangements can lead to dangerous research practices or deliberate misuse, potentially resulting in severe threats to global health and security. Additionally, this scheme does not remove the ethical burden from the shoulders of Country B. On the contrary, this form of collaboration could build a sense of being shielded from scrutiny in Country B, thus encouraging Country B to not uphold its international responsibilities in limiting the development of biological weapons.

### Country B Motivations

In this speculative exploration, we delve into a hypothetical scenario involving two powerful nations, Country A and Country B, where one enjoys a higher GDP and greater financial resources, while the other experiences rapid economic growth, bolstered by a larger population and notable biotechnological expertise. In the midst of their covert power struggle, Country A decides to fund Country B’s research and development of viruses with potential use as bioweapons, prompting us to examine the possible reasons behind this collaboration. Within this fictional context, we identify various hypothetical points that may explain Country B’s motivations for accepting this arrangement. As we traverse this speculative landscape, it’s essential to remember that these points are entirely fictional, designed for academic exploration. Throughout this analysis, we remain mindful of the ethical concerns and global health security risks associated with bioweapons research and the implications for international scientific cooperation.

Country B’s acceptance of collaboration and funding from Country A could be driven by the potential economic advantages it presents. With financial resources from Country A, Country B can make significant investments in its biotechnological infrastructure, fostering economic growth and innovation (Fan 2011). The influx of funds could enable Country B to build state-of-the-art research facilities, providing a cutting-edge environment for bioweapons research. Additionally, Country B could use this capital to attract highly skilled scientists and procure advanced technology, helping to position itself as a regional hub for biotechnological innovation. These developments can have a ripple effect on Country B’s economy, encouraging broader industrial growth and job creation. The presence of advanced research facilities could attract interest from private industry, leading to new business partnerships and collaboration opportunities. Furthermore, with Country B emerging as a key player in bioweapons research, international investors may see this as a lucrative market, further driving economic development. This could result in a surge of foreign direct investment, fostering new industries and boosting overall economic prosperity in Country B.

Country B’s collaboration with Country A could provide an opportunity to gain access to advanced expertise and knowledge in bioweapons research. Working alongside Country A’s leading scientists, Country B can learn from cutting-edge methodologies and expand its own scientific capabilities (Flink and Schreiterer 2010). This collaboration can lead to a more comprehensive understanding of bioweapons development, enabling Country B to achieve breakthroughs that would otherwise be difficult to attain independently. The increased scientific knowledge can accelerate Country B’s research, leading to a rise in publications and recognition within the scientific community. Country B can strengthen its biodefense, enhancing its ability to counter biological threats. This partnership might enable the development of vaccines, antidotes, and other protective measures, significantly improving Country B’s readiness to handle potential bioweapons attacks.

The partnership with Country A can significantly boost Country B’s global visibility in the scientific and biotechnological landscape. As Country A is widely recognized for its expertise in bioweapons research, its collaboration with Country B lends credibility and recognition to Country B’s efforts. This partnership can draw international attention to Country B’s research endeavours, opening doors to invitations for global scientific conferences, symposia, and other collaborative projects (Dua et al. 2023; Bhattacharya and Kaul 2015). This enhanced visibility can lead to additional research funding from international sources, enabling Country B to expand its research initiatives and attract top talent from around the world. As Country B gains recognition on the global stage, it can also build strategic alliances with other countries, fostering a network of international partnerships that can further strengthen its position in the scientific community.

Country B’s willingness to collaborate with Country A could be part of a broader strategy to strengthen strategic alliances. By engaging in joint bioweapons research, Country B can demonstrate its commitment to fostering deeper cooperation and mutual support with Country A. This collaboration can be viewed as a signal of trust and shared objectives, helping to build a more robust diplomatic relationship between the two countries (Vijayalakshmi 2017). The strategic alliance created through this collaboration could lead to additional benefits for Country B, such as gaining political leverage, preferential trade agreements, security partnerships, or joint initiatives in other areas of mutual interest. By working together on bioweapons research, Country B and Country A can align their strategic interests, promoting stability and cooperation. This collaboration could also serve as a foundation for future projects, reinforcing the alliance and ensuring ongoing support from Country A. However, such collaborations may raise geopolitical concerns, especially if Country B seeks alliances with other nations that are less aligned with Country A’s interests. An example of this is India’s involvement in the BRICS agreement, which has caused notable discontent among U.S. policymakers due to perceived misalignment with their geopolitical strategies (Lalwani and Byrne 2019).

Country B’s participation in bioweapons research could be driven by a sense of national pride and prestige. By engaging in advanced biotechnological research, Country B can showcase its scientific prowess, demonstrating its ability to contribute to cutting-edge fields. The achievements in bioweapons research could become a source of national pride, celebrated as a sign of technological and scientific excellence. This enhanced prestige can elevate Country B’s status and influence on the international stage, positioning it as a key player in global bioweapons research and related domains. This sense of national pride can have broader implications, fostering a sense of unity and confidence within Country B (Hall 2016). The recognition of scientific achievements can inspire future generations to pursue careers in science and technology, leading to a more robust and innovative workforce. Additionally, the increased prestige can help Country B gain international respect, promoting its image as a nation capable of significant contributions to global health security and beyond. This enhanced status can open doors to new opportunities for collaboration, further strengthening Country B’s influence in the global scientific community.

## Discussion

**Fig. 2:**
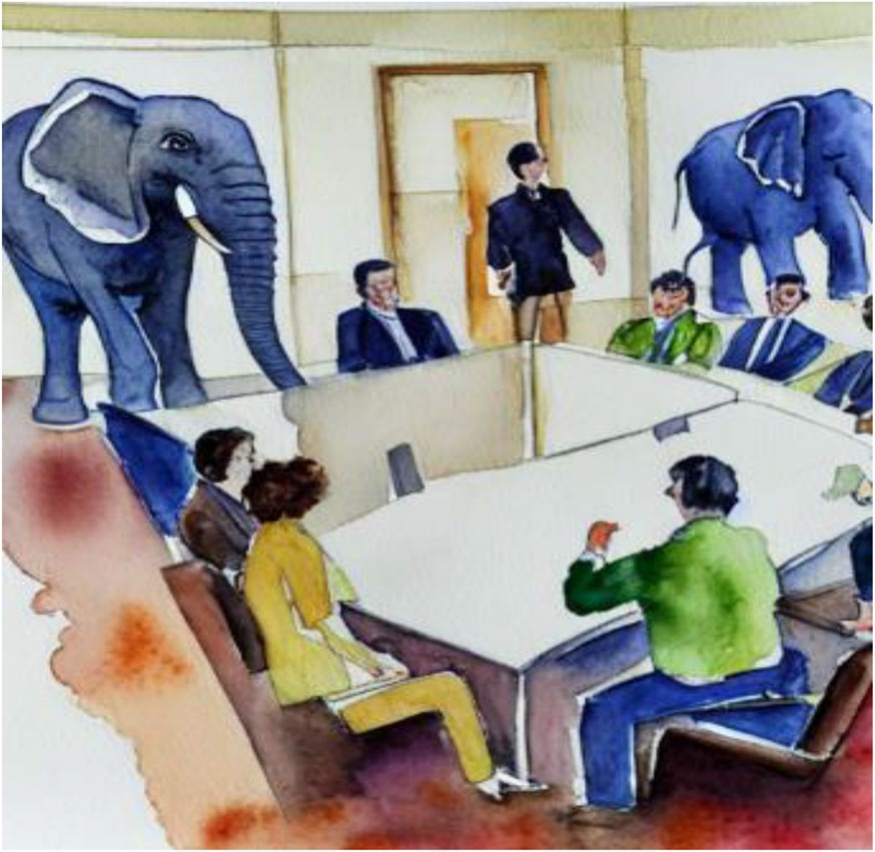
An elephant in the room situation. The scientific community is ignoring the geopolitical struggle between superpowers when it comes to the bioethics of bioweapons research. (Figure created by AI)

In this hypothetical exploration, we examine potential motivations for Country A to fund Country B’s research into bioweapons and consider reasons for Country B’s acceptance of such collaboration. This scenario illuminates the complexities and implications of bioweapons research in the context of geopolitics, national interests, and ethical considerations, and it highlights a significant international issue that could shape the trajectory of scientific development. However, this issue is often ignored, resembling the classic “elephant in the room” (Fig. 2) (Churchill et al. 2020).

Country A might fund Country B’s bioweapons research to gain geopolitical leverage, create dependencies, gather bio-intelligence, generate revenue from innovations, or use proxies to conduct research that would face backlash at home. Country B, in turn, may use this opportunity for scientific and economic benefits, boosting national pride and visibility. India’s Prime Minister Modi, for example, has championed initiatives to enhance India’s global stature, leverage international partnerships for development, hedge against China’s influence, safeguard national interests, and mobilize the Indian diaspora for strategic goals (Hall 2016). However, such partnerships raise ethical concerns. The drive for scientific progress must be balanced with global health security, requiring that ethical considerations guide bioweapons research to ensure public safety is not compromised.

In the wake of ongoing debates about the origins of COVID-19, the focus should shift toward bioethics rather than blame. While uncovering the true origins of the virus is crucial for prevention and future readiness, a relentless pursuit of blame could hamper constructive efforts (van Helden et al. 20,212). Ethical principles guide responsible research, promote transparency, and foster international collaboration, leading to effective solutions for public health challenges. Emphasizing bioethics encourages open dialogue and responsible data sharing, building trust among nations and facilitating a united response to health crises.

The hypothetical knowledge derived from this exploration of bioweapons research collaboration can be leveraged to improve biosecurity in several ways. The insights gained underscore the importance of international cooperation in developing comprehensive biosecurity frameworks. Collaborating countries can create clear guidelines for strengthening monitoring of potentially sensitive bioweapons research, ensuring that biosafety concerns are addressed. Transparent communication and data sharing between countries are crucial. By promoting open exchange, countries can collectively tackle biosafety challenges, share best practices, and manage the risks associated with bioweapons research. To ensure accountability, there could be systems to penalize countries engaging in illicit bioweapons research (Shearer et al. 2022). Strengthening the United Nations’ role could reduce exploitation of poorer countries by more powerful ones, promoting equitable biosecurity (Elnaiem et al. 2023).

## References

[CR1] Akhtar, R. 2023. Inheriting the bomb: The collapse of the USSR and the nuclear disarmament of Ukraine. *International Affairs* 99(5):2174–2175.

[CR2] Anderson, P.D. and G. Bokor. 2012. Bioterrorism: Pathogens as weapons. *Journal of Pharmaceutical Practice* 25(5): 521–529. 10.1177/0897190012456366 23011963

[CR3] Bhattacharya, S.S., and A. Kaul. 2015. Emerging countries assertion in the global publication landscape of science: A case study of India. *Scientometrics* 103: 387–411.

[CR4] Churchill, L.R., N.M.P. King, and G.E. Henderson. 2020. The future of bioethics: It shouldn’t take a pandemic. *Hastings Center Report* 50(3): 54–56. 32596911 10.1002/hast.1133

[CR5] Dua, J., V.K. Singh, and H.H. Lathabai. 2023. Measuring and characterizing international collaboration patterns in Indian scientific research. *Scientometrics* 128(9): 5081–5116.

[CR6] Elnaiem, A., and O. Mohamed-Ahmed, A. Zumla, et al. 2023. Global and regional governance of One Health and implications for global health security. *Lancet* 401(10377): 688–704. 36682375 10.1016/S0140-6736(22)01597-5

[CR7] Fan, P. 2011. Innovation capacity and economic development: China and India. *Economic Change and Restructuring* 44: 49–73.

[CR8] Flink, T., and U. Schreiterer. 2010. Science diplomacy at the intersection of S&T policies and foreign affairs: Toward a typology of national approaches. *Science and Public Policy* 37(9): 665–677.

[CR9] Geiger, S., and N. Gross. 2024. Tech sharing, not tech hoarding: Covid-19, global solidarity, and the failed responsibility of the pharmaceutical industry. *Organization* 31(3): 567–582.

[CR10] Giffard, H. 2020. Exploiting Nazi science and technology and the history of technology transfer. *Historical Studies in the Natural Science* 50(1–2): 199–208.

[CR11] Hall, I. 2016. Multialignment and Indian foreign policy under Narendra Modi. *Round Table* 105(3): 271–286.

[CR12] Huigang, L., L. Menghui, Z. Xiaoli, H. Cui, and Y. Zhiming. 2022. Development of and prospects for the biological weapons convention. *Journal of Biosafety and Biosecurity* 4(1): 50–53.

[CR13] Johnson, K. 2022. A scientific method to the madness of Unit 731’s uman Experimentation and Biological Warfare Program. *Journal of the History of Medicine and Allied Sciences* 77(1): 24–47. 34897467 10.1093/jhmas/jrab044

[CR14] Johnson, T.F. 2024. For the good of the globe: Moral reasons for states to mitigate global catastrophic biological risks. *Journal of Bioethical Inquiry*, Epub ahead of print. 10.1007/s11673-024-10337-z. 10.1007/s11673-024-10337-z PMC11652646 38329644

[CR15] Kadlec, R.P., A.P. Zelicoff, and A.M. Vrtis. 1997. Biological weapons control: Prospects and implications for the future. *JAMA* 278(5): 351–356. 9244311 10.1001/jama.278.5.351

[CR16] Lalwani, S., and H. Byrne. 2019. Great expectations: Asking too much of the U.S.–India strategic partnership. *Washington Quarterly* 42(3): 41–64.

[CR17] Li, P. 2017. Japan’s biochemical warfare and experimentation in China. In *Japanese War Crimes*, edited by P. Li, 289–300. Routledge.

[CR18] Meng, B. 2017. The historical significance of the biological weapons convention (BWC). *SSRN Electronics Journal*. 10.2139/ssrn.3067204.

[CR19] Molm, L.D. 2015. Power‐dependence theory. In *The Blackwell Encyclopedia of Sociology*. Wiley.

[CR20] Nealey, T., R.M. Daignault, and Y. Cai. 2015. Trade secrets in life science and pharmaceutical companies. *Cold Spring Harbour Perspectives in Medicine* 5(4): a020982. 10.1101/cshperspect.a020982 PMC4382727 25414378

[CR21] Nelson, M.I., J.O. Lloyd-Smith, L. Simonsen, et al. 2019. Fogarty International Center Collaborative Networks in Infectious Disease Modeling: Lessons learnt in research and capacity building. *Epidemics* 26: 116–127. 30446431 10.1016/j.epidem.2018.10.004 PMC7105018

[CR22] Ouagrham-Gormley, S.B. 2012. Barriers to bioweapons: Intangible obstacles to proliferation. *International Security* 36(4): 80–114.

[CR23] Roberts, P. 2012. *Voices of World War II: Contemporary accounts of daily life*. Bloomsbury Publishing.

[CR24] Roffey, R. 2015. The Soviet biological weapons program: A history [book review]. *Contemporary Security Policy* 36(2): 401–404.

[CR25] Shearer, M.P., C. Potter, R.A. Vahey, N.D. Connell, and G.K. Gronvall. 2022. BWC assurance: Increasing certainty in BWC compliance. *Nonproliferation Review* 29(1–3): 47–75.

[CR26] Sims, N.A. 2007. The future of biological disarmament. *Nonproliferation Review* 14(2): 351–372.

[CR27] van Helden, J., C.D. Butler, G. Achaz, et al. 2021. An appeal for an objective, open, and transparent scientific debate about the origin of SARS-CoV-2. *Lancet* 398(10309): 1402–1404. 34543608 10.1016/S0140-6736(21)02019-5 PMC8448488

[CR28] Vijayalakshmi, K.P. 2017. India–U.S. strategic partnership: Shifting American perspectives on engaging India. *International Studies* 54(1–4): 42–61.

[CR29] Wissinger, J. 2015. The BWC gray area: Locating the blurry line of defining biological weapons. *Journal of Biosecurity, Biosafety, and Biodefense Law* 6(1): 1–21.

[CR30] Zellman, A. 2014. Proxy warfare. *Political Science Quarterly* 129(2): 352–354.

